# Continuous Feedings of Fortified Human Milk Lead to Nutrient Losses of Fat, Calcium and Phosphorous

**DOI:** 10.3390/nu2030240

**Published:** 2010-02-26

**Authors:** Stefanie P. Rogers, Penni D. Hicks, Maria Hamzo, Lauren E. Veit, Steven A. Abrams

**Affiliations:** 1 Northwest Newborn Specialists 501 N, Graham Suite 265, Portland, OR 97227 USA; Email: stefanie@nwnewborn.com; 2 United States Department of Agriculture/Agricultural Research Service, Children's Nutrition Research Center, Department of Pediatrics, Baylor College of Medicine, 1100 Bates St. Houston, Texas 77030 USA; Email: hamzom@stthom.edu (M.H.); pennih@bcm.edu(P.D.H.); 3 Department of Biomedical Engineering, Rensselaer Polytechnic Institute, Troy, NY 12180 USA; Email: Veitl@rpi.edu; 4 Section of Neonatology, Department of Pediatrics, Baylor College of Medicine and Texas Children’s Hospital, Houston, Texas 77030 USA

**Keywords:** calcium, phosphorous, neonates, enteral nutrition, lipids, protein

## Abstract

Substantial losses of nutrients may occur during tube (gavage) feeding of fortified human milk. Our objective was to compare the losses of key macronutrients and minerals based on method of fortification and gavage feeding method. We used clinically available gavage feeding systems and measured pre- and post-feeding (end-point) nutrient content of calcium (Ca), phosphorus (Phos), protein, and fat. Comparisons were made between continuous, gravity bolus, and 30-minute infusion pump feeding systems, as well as human milk fortified with donor human milk-based and bovine milk-based human milk fortifier using an in vitro model. Feeding method was significantly associated with fat and Ca losses, with increased losses in continuous feeds. Fat losses in continuous feeds were substantial, with 40 ± 3 % of initial fat lost during the feeding process. After correction for feeding method, human milk fortified with donor milk-based fortifier was associated with significantly less loss of Ca (8 ± 4% *vs.* 28 ± 4%, p< 0.001), Phos (3 ± 4% *vs.* 24 ± 4%, p < 0.001), and fat (17 ± 2% *vs.* 25 ± 2%, p = 0.001) than human milk fortified with a bovine milk-based fortifier (Mean ± SEM).

## 1. Introduction

Human milk is the recommended feeding for nearly all infants including very low birth weight (VLBW) infants. VLBW infants fed human milk, especially their own mother’s milk, have lower rates of infection and necrotizing enterocolitis, improved neurologic outcome, and improved feeding tolerance [1,2,3,4,5,6,7]. Although human milk is optimal for VLBW infants, it is necessary to fortify it with macronutrients and minerals in order to meet the nutritional needs of premature infants [8]. Currently, powdered bovine milk-based fortifiers are used in most neonatal intensive care units (NICU). Prolacta Bioscience (Monrovia, CA) has begun marketing human milk-based nutritional products to preterm infants in the US. The company produces both donor milk and a fortifier made from donated milk designed to meet the macronutrient and micronutrient needs of preterm infants [9]. Recently, there has been increasing use of this fortifier which only contains human milk proteins with the goal of improved feeding tolerance. The fortifier can be added to donor milk or mother’s own milk as a high calorie, high mineral-containing liquid. 

Regardless of the fortifier used, fortified human milk (FHM) is usually delivered via gavage until the infant is able to feed orally. Milk provided by gavage feeding may be delivered via a variety of methods. These include gravity bolus, in which the feedings are allowed to drip over 10–15 minutes into the infant; via infusion using an infusion pump set to deliver the milk over a set time, typically 30-60 minutes; or via continuous feeding, using an infusion pump or commercially available roller head pump. Each of these methods is used widely and often one infant will receive multiple approaches to feeding during their hospitalization. There are limited data to indicate that any one method is superior to another. Use of any of these methods may lead to substantial loss of nutrients. Limited data related to feeding method and nutrient losses were published previously, but these older studies do not reflect current feeding and delivery systems [10,11,12,13,14,15]. There have been no recent publications evaluating nutrient losses. In general, nutrient intake calculations as performed in a NICU setting do not account for the nutrient losses associated with gavage feeding. Knowledge of the loss of nutrients incurred during the gavage process is necessary in order to accurately assess nutrient intake and the effect of nutritional interventions.

Our NICU has begun use of human milk-based fortifier as a part of research protocols. Some of the staff have noted that there is less visible separation of human milk and adherence to the delivery system when using these products. This encouraged us to study if nutrient delivery is different in human milk fortified with human milk-based fortifiers and bovine milk-based fortifiers, and how the delivery systems affect nutrient delivery. Therefore, we sought to clarify the nutrient losses that occur during the delivery process of fortified human milk by creating in vitro a tube feeding system that simulates the usual NICU feeding methods in our unit. With this system we assessed the nutrient losses from fortified human milk of different forms and via different feeding approaches. We evaluated losses of key nutrients: fat, protein, calcium, and phosphorous. Fat and Protein were chosen as adequate protein and calorie intake is key to growth (fat is the main contributor to total calories) [16]. Calcium and phosphorous were chosen as these minerals often complex with fat and deficiencies can lead to osteopenia or rickets in preterm infants [17].

Our primary aim was to compare the losses of key macronutrients and minerals based on method of fortification and gavage feeding method in infants fed fortified human milk. Secondary aims were to provide information useful to assess actual nutrient intakes and to provide a basis for determining potential needs for improvement in milk delivery systems.

## 2. Experimental Section

### 2.1. Simulated Feedings

We evaluated the use of both human milk fortified with donor human milk-based fortifier (HM-DonF) and human milk fortified with bovine milk-based fortifier (HM-BovF) using an in vitro model of stimulated feedings. Banked, unprocessed donor human milk (Prolacta Bioscience, Monrovia, CA) stored at −20 ^o^C was thawed and then mixed with either bovine-based human milk fortifier (Similac Human Milk Fortifier, Abbott Laboratories, Columbus, OH) or donor human milk-based human milk fortifier (Prolact +4 H2MF, Prolacta Bioscience, Monrovia, CA) per manufacturers instructions to achieve approximately 81 kcal/100 mL final concentration. All mothers were human milk donors prior to study inception and had given written informed consent for use of their milk for consumption and/or general research purposes upon donation to Prolacta Bioscience. Institutional Review Board approval was not necessary because only pooled, non-identifiable milk samples were used in this study. The banked, unprocessed donor milk was frozen and should be considered equivalent to mother’s milk provided to infants. 

FHM was separated into aliquots. A “pre” feeding aliquot of 30 mL was immediately processed after mixing for analysis of calcium (Ca), phosphorous (Phos), protein, and fat. To simulate conditions in the NICU where feedings are often stored after mixing, a second 30 mL aliquot was stored overnight (8–16 hours) at 4 ^o^C, then warmed in a 40 ^o^C water bath and given as a simulated feeding. All simulated feeds were given through a 5 French, 90 cm orogastric feeding tube (Kendall Argyle, Tyco Healthcare, Mansfield, MA). The simulated feeding was collected in its entirety and analyzed for Ca, Phos, protein and fat as a “post” aliquot. Nutrient loss was expressed as a percentage of initial nutrient concentration. 

Simulated feeding conditions included: bolus by gravity at height of 30 cm (n = 15 for each fortifier), bolus over 30 minutes by infusion pump (Medfusion 2010, Medex Inc, Duluth, GA) (n = 5 for each fortifier), and continuous delivery over 3 hours with a commercially available roller head pump (Kangaroo 324 and Kangaroo Epump, Sherwood Medical, St.Louis, MO) (n = 15 for each fortifier). Boluses given by gravity feeds were oriented in the vertical direction. Boluses given over 30 minutes by infusion pump feeds were infused at a rate of 60 mL/hr and were oriented in the horizontal direction. Continuous infusion feeds ran at a rate of 10 mL/hr. 

The simulated continuous feedings were given through Kangaroo Pump Feeding Bags and tubing (with the Kangaroo 324) (n = 5 for each fortifier) or Kangaroo Epump 100 mL Burette Set (with the Kangaroo Epump) (n = 10 for each fortifier) in addition to the 5 French orogastric feeding tube. A new set up was used for each simulated feed. During the process of the data collection, our hospital stopped using the Kanagaroo 324, and transitioned to the Kangaroo Epump for delivery of continuous feeds. Data were collected on both systems. 

### 2.2. Nutrient Analysis

#### 2.2.1. Calcium and Phosphorous

FHM aliquots were microwave digested in HNO3 acid, dried and reconstituted with 20 mL of ultrapure 0.1N HNO3. Samples were then analyzed in triplicate for total minerals using inductively coupled plasma analysis. Average values were determined and used for calculations. Precision and accuracy of this method is <5%.

#### 2.2.2. Fat and Protein

FHM aliquots of both donor human milk-based and bovine milk- based fortifier were analyzed by the supplier of the donor human milk using mid-range infrared analysis using an Acudairy 5150 (ATI Inc., Westfield, NY) that has been calibrated for use with human milk. Precision and accuracy of this method is <5%. 

### 2.3. Statistical Analysis

Data were analyzed using SPSS (Version 16, Chicago, Il) and the relationships among nutrient losses and other outcome variables evaluated by general linear analysis. All data are presented as mean ± SEM using pooled variances from the analysis. The interaction of feeding type and delivery method was evaluated. If this interaction was significant (p < 0.05), then analysis was performed separately for each feeding type. Pair-wise comparisons were performed for significant comparisons (p < 0.05) identified in the analysis. 

## 3. Results and Discussion

Table 1Nutrient Composition of 100 mL Fortified Human Milk Prior to Simulated Feeds.

**Table:** A. HM-DonF

	Mean	SEM	Per Label^18 ^
Ca (mg)	99.8	4.8	94
Phos (mg)	60.1	0.7	44
Fat (g)	3.7	0.2	4.8
Protein (g)	2.1	0.03	2

Values shown reflect HM-DonF at time of study. Mineral composition has been increased to 112 mg Ca and 53 mg Phos per 100 mL fortified human milk.

**Table:** B. HM-BovF

	Mean	SEM	Per Label^19 ^
Ca (mg)	165.4	7.6	138
Phos (mg)	100.3	3.3	78
Fat (g)	3.1	0.2	4.1
Protein (g)	2.2	0.04	2.3

Table 2Endpoint Nutrient Concentration by Fortifier and feeding method (Mean ± SEM).

**Table:** A. HM-DonF

	*Gravity*	*Infusion*	*Continuous 324*	*Continuous Epump*
Ca (mg)	96.3 ± 3.3	108.8 ± 1.9	95.1 ± 2.8	87.6 ± 2.5
Phos (mg)	60.1 ± 0.7	57.5 ± 0.5	57.3 ± 0.8	60.9 ± 1.1
Fat (g)	3.5 ± 0.2	2.9 ± 0.4	2.2 ± 0.3	3.2 ± 0.1
Protein (g)	2.1 ± 0	2.0 ± 0.1	2.0 ± 0	2.1 ± 0

**Table:** B. HM-BovF

	***Gravity***	***Infusion***	***Continuous 324***	***Continuous Epump***
Ca (mg)	137 ± 11.8	110.3 ± 4.0	62.4 ± 16.9	119.4 ± 17.3
Phos (mg)	84.9 ± 6.1	68.6 ± 3.4	64.5 ± 32.2	77.6 ± 8.7
Fat (g)	2.9 ± 0.2	2.4 ± 0.5	1.4 ± 0.3	2.3 ± 0.1
Protein (g)	2.3 ± 0	2.1 ± 0.1	2.1 ± 0	2.2 ± 0

**Table 3:** Percent nutrient losses by fortifier.

	HM-DonF	HM-BovF	*p*-value
**Number of samples**	35	35	
Ca (%)	8 ± 4	28 ± 4	<0.001
Phos (%)	3 ± 4	24 ± 4	<0.001
Fat (%)	17 ± 2	25 ±2	0.001
Protein (%)	0 ± 1	−1 ± 1	0.56

Values shown are corrected for feeding method. There was no significant interaction of feeding method and fortifier type. Data shown are Mean ± SEM using the pooled SEM from ANOVA analysis.

**Table 4:** Percent nutrient losses by feeding mechanism.

	Infusion via gravity	Infusion via pump	Continuous with 324	Continuous with Epump	*p*-value
**Number of samples**	30	10	10	20	
Ca (%)	9 ± 4	14 ± 7	33 ± 7	17 ± 5	0.04*
Phos (%)	7 ± 4	14 ± 6	20 ± 6	12 ± 4	0.35
Fat (%)	6 ± 2	13 ± 3	40 ± 3	25 ± 2	<0.001**
Protein (%)	−3 ± 1	0 ± 1	1 ± 2	−1 ± 1	0.10

Values shown are corrected for fortifier type. There was no significant interaction of feeding method and milk type. Data shown are Mean ± SEM using the pooled SEM from ANOVA analysis. *Interaction term of feeding method and milk type not significant, p = 0.57. Paired difference of Grav *vs.* Cont 324 was significant, p = 0.005, all others p > 0.05. **interaction term of feeding method and milk type significant, p = 0.02. All paired comparisons are significant, P < 0.01 except for Grav *vs.* Infusion which is p = 0.06.

**Table 5:** Percent fat loss differences based on feeding type and fortification source.

	HM-DonF	HM-BovF	*p*-value
**Number of samples**	35	35	
Infusion via gravity (n = 15 each)	6 ± 2	6 ± 2	0.83
Infusion via pump (n = 5 each)	10 ± 2	16 ± 2	0.10
Continuous with 324 (n = 5 each)	29 ± 4	51 ± 4	0.007
Continuous with Epump (n = 10 each)	22 ± 4	28 ± 4	0.22

**Table 6:** Percent nutrient losses in continuous feeds

	Continuous with 324	Continuous with Epump	*p*-value
**Number of samples**	10	20	
Ca (%)	33 ± 8	17 ± 5	0.10
Phos (%)	20 ± 7	12 ± 5	0.39
Fat (%)	40 ± 3	25 ± 3	0.003*

*Interaction term of feeding method and milk type, p = 0.08. Analysis performed including this term. Interaction term NS for Ca and Phos and excluded from analysis.

**Figure 1:**
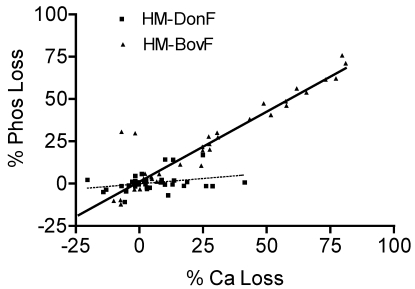
HM-BovF shows a significant linear relationship for Ca and Phos losses. This is not seen for HM-DonF.

Table 1 shows the nutrient composition of fortified human milk prior to any simulated feeds. Table 3 shows the percent nutrient losses in the different fortifiers corrected for feeding method. A substantial difference between fortifiers was seen for Ca and Phos, with a smaller but also statistically significant difference seen for fat. Protein content changes were minimal and were essentially identical between fortifiers. 

Nutrient losses, as related to feeding mechanism, are shown in Table 4. All nutrients were maintained at higher levels with more rapid infusions. This was significant for Ca and fat, but did not reach significance for Phos. Protein losses were again minimal and not significantly different among the feeding mechanisms. In pairwise comparisons, Ca losses were significantly lower using gravity compared to continuous feeds (p < 0.05). 

A significant interaction was seen for fat losses between feeding method and fortifier type (p = 0.02). We, therefore, evaluated the effects of specific feeding methods on the difference seen in human milk fortified with donor milk-based fortifier *vs.* human milk fortified with bovine milk-based fortifier for fat loss. This showed (Table 5) that the significant effect was largely related to the Kangaroo 324 continuous pump having very high fat losses. Of importance to note is that no differences in fat losses between fortifiers were seen using Gravity bolus feeds.

We evaluated both the Kangaroo 324 and Kangaroo Epump. The use of the newer Epump markedly decreased the loss of fat (Table 6). There were also reduced Ca and Phos losses, but this did not achieve statistical significance due to the greater variability in the mineral results. There was no difference between the protein losses. 

As shown in Figure 1, a significant linear relationship between Ca and Phos losses was seen overall in fortified human milk (r = 0.88, p < 0.001), suggesting direct loss of calcium phosphate salts. However, when examining the type of fortifier used, this linear relationship remained significant only for HM-BovF (r = 0.92, p < 0.001), but not HM-DonF (r = 0.3, p = 0.09). There was no evidence of a relationship between Ca and fat losses overall (r = 0.1, p = 0.3) or in HM-DonF (r = 0.1, p = 0.5) or HM-BovF (r = 0.03, p = 0.8), further suggesting the loss of calcium primarily as bound to phosphate. 

## 4. Discussion and Conclusions

We found substantial losses of key minerals and fat, but not protein, associated with the feeding of fortified human milk by gavage. Factors that minimized these losses were the use of liquid donor human milk-based fortifier, the use of bolus or infusion over 30 minutes instead of continuous feeds, and the use of a newer pump system for the delivery of continuous feeds. We hypothesize that nutrient losses could be due to improper mixing, precipitation, adherence to the delivery systems or other unknown factors.

This study confirms that nutrient losses during routine feeding practices in the nursery are still a concern and remain similar to those seen in older studies, despite improvements and enhancements of human milk fortifiers. This is the first study to evaluate both macronutrient and mineral losses that occur in fortified human milk. Furthermore, this is the first comparison of a human milk-based fortifier with traditional bovine milk-based fortifier. 

In evaluating the impact of these losses on caloric intake, the effect ranged from as little as 6% of fat lost when feeds were given via bolus by gravity to up to 50% of fat lost when powdered bovine milk-based fortified human milk was given by continuous feeding using an older pump. Assuming approximately 50% calories from fat and intake targets of 120 kcal/kg/d, this would represent from 3 kcal/kg/d to about 25 kcal/kg/d lost in the feeding. It is important to note that the smallest of infants often do not tolerate gravity bolus feedings and feedings are given by gavage over at least 30 minutes or by continuous gavage. Our data indicate that this practice may affect the nutrient delivery to these vulnerable infants. 

The reason for the significant improvement in fat losses we observed in continuous feeds with the Kangaroo Epump compared to the Kangaroo 324 is uncertain. Both pumps use a rotary peristaltic method for delivery of feedings. However, the Epump does not have a collection chamber and has shorter tubing length. We hypothesize that these factors may have been the reason for significantly reduced losses, but further evaluation is needed. 

The linear relationship between Ca and Phos losses was highly significant in human milk fortified with a bovine milk-based fortifier with a slope approximating a mg:mg loss. This suggests direct loss of calcium phosphate salts in HM-BovF. The mineral losses with fortified human milk indicate that the primary issue is likely to be insolubility and adherence in the tubing delivery system. There was no evidence of a significant relationship between Ca and fat losses in fortified human milk indicating that the losses of Ca are primarily related to added calcium salts rather than the intrinsic human milk calcium. 

Previous research also shows marked losses of nutrients from human milk, especially lipids, when delivered through simulated feeds [10,11,12,13,14]. However, those studies were all performed over 20 years ago and used exclusively unfortified human milk. Brooke (1978) was the first to show losses during continuous feeds with variations up to 24% in fat content with recovery of much of the fat content in residue and washings of tubing [10]. Using slower infusions and the use of continuous feedings exacerbate these losses as demonstrated by Greer in 1984 [11]. Furthermore, Greer showed decreased losses with the syringe pump in the vertical position for moderate and fast infusion times. Both Greer and Narayanan (1984) found an energy rich aliquot at the end of the feed in continuous feeds, but Narayanan did not see this with intermittent feeds [12]. Narayanan hypothesized that fat losses were minimized by an eccentric nozzle. Stocks (1985) confirmed an inverse correlation between fat loss and flow rates [13]. Of note, Stocks evaluated protein losses and found them to be 7% for bolus and 5% for continuous feeds. This is in contrast to our relative sparing of protein. We believe the “gain” of protein we saw was due to a relative loss of water. All authors cite visible separation of the milk and adherence to the tubing as the reason for the substantial losses. There has been a paucity of investigation into nutrient losses during gavage feedings since these articles were published, and despite improvements in fortifiers over the last 20 years substantial nutrient losses can still be seen.

Bhatia evaluated mineral losses from premature infant formulas [15]. He found that bolus feeds minimized mineral losses in formula (Ca losses were 0–9%, and Phos losses were relative gain of 2 to a loss of 8%). Depending on the formula, 25–41% of the calcium was lost, and relative gain of 2% to loss of 33% of the phosphorous was seen in continuous feeds. To our knowledge, there are no previous studies that examine the mineral losses encountered during routine nursery feeding practices of human milk. Our results in fortified human milk indicate that approx 9% of Ca is lost and 7% of Phos is lost in bolus by gravity feeds and 17–33% of Ca is lost, and 12–20% of Phos is lost in continuous feeds. These numbers are similar to the losses seen in formula. The majority of the Ca and Phos in fortified human milk are supplied by the fortifier. Since many of the salts that are added to the fortifiers are the same salts that are added to preterm formula, these results are not surprising. 

Our primary aim was to compare the losses of nutrients based on method of fortification and gavage feeding method. We strove to determine if any substantial improvement in nutrient losses has occurred in the last 20 years. In an attempt to be consistent with previous studies, we reported the data in terms of percent loss. Use of percent loss is also helpful as the donor milk based fortifier product recently increased their mineral concentration. We performed the study on the older formulation. Mineral composition has been increased to 112 mg Ca and 53 mg Phos per 100 mL fortified human milk. It is possible that this change in mineral levels could affect losses, but since a direct loss of calcium phosphate salts was not seen in HM-DonF, we believe this is unlikely. Furthermore, the donor milk based fortifier uses the same calcium salts and emulsifiers as were used prior to the reformulation, leading to minimal change in percentage loss. However the use of percent loss may mask the true final concentration of nutrients that are present. For this reason we have included the initial and endpoint nutrient concentrations. 

This study did not evaluate other losses, nutritive and non-nutritive in nature (e.g., vitamins, immunoglobins, *etc.*). Additionally, we did not look for a bolus of fat delivered at the end of the feeding, or for the ability to recover nutrients from the feeding syringe or tubing. However, our study design was intended to simulate actual feeding conditions and therefore post-feeding recovery of nutrients was not our goal. An additional limitation is that a new feeding tube was used for each simulated feed. In clinical practice indwelling gavage tubes are often used for more than one feed. The nutrient losses seen in our study may not represent the losses seen over the life of the feeding tube. 

Nutrient losses from delivery method and fortifier type may be contributing factors to poor growth in the human milk fed neonate. The substantial losses that we have shown during the gavage feeding process indicate that we still may not be delivering feeds that meet the nutrient needs of premature infants; this is especially true for continuous feedings of fortified human milk. Further research is necessary to investigate factors that limit these losses. Although some infants may tolerate feedings better when given continuously, evidence to support the routine use of this practice in VLBW infants is minimal [20,21]. In deciding on nursery practices, the loss of nutrients during continuous feedings of fortified human milk should be considered. Nutrient intake calculations may need to reflect these losses and consideration should be given to avoiding only using long-term continuous feeds in infants who are at nutritional risk.
